# The Hidden Enemy Within: Non-canonical Peptides in Virus-Induced Autoimmunity

**DOI:** 10.3389/fmicb.2022.840911

**Published:** 2022-02-10

**Authors:** Manivel Lodha, Florian Erhard, Lars Dölken, Bhupesh K. Prusty

**Affiliations:** Institute for Virology and Immunobiology, Julius-Maximilians University of Würzburg, Würzburg, Germany

**Keywords:** viruses, cryptic peptides, autoimmunity, defective ribosomal products, non-canonical translation, COVID-19

## Abstract

Viruses play a key role in explaining the pathogenesis of various autoimmune disorders, whose underlying principle is defined by the activation of autoreactive T-cells. In many cases, T-cells escape self-tolerance due to the failure in encountering certain MHC-I self-peptide complexes at substantial levels, whose peptides remain invisible from the immune system. Over the years, contribution of unstable defective ribosomal products (DRiPs) in immunosurveillance has gained prominence. A class of unstable products emerge from non-canonical translation and processing of unannotated mammalian and viral ORFs and their peptides are cryptic in nature. Indeed, high throughput sequencing and proteomics have revealed that a substantial portion of our genomes comprise of non-canonical ORFs, whose generation is significantly modulated during disease. Many of these ORFs comprise short ORFs (sORFs) and upstream ORFs (uORFs) that resemble DRiPs and may hence be preferentially presented. Here, we discuss how such products, normally “hidden” from the immune system, become abundant in viral infections activating autoimmune T-cells, by discussing their emerging role in infection and disease. Finally, we provide a perspective on how these mechanisms can explain several autoimmune disorders in the wake of the COVID-19 pandemic.

## Introduction

One of the salient features of autoimmunity lies in the recognition of MHC class I associated self-peptides by T-cells that have escaped self-tolerance during their development and selection in the thymus. Self-peptides, not generated and presented at substantial levels for T-cells to undergo anergy and deletion, can cause T-cells to escape selection. Such cryptic peptides might be generated and presented under certain stimuli to activate self-specific T-cells, forming the fundamental basis of autoimmunity (Sercarz et al., 1993; Lanzavecchia, 1995). Over the years, many extrinsic factors including viral infections have served to explain autoimmunity through the generation of self-peptide mimics, bystander activation of T-cells and epitope spreading (Smatti et al., 2019). However, the exact nature and generation of such peptides remains loosely defined, hampering the development of broad range therapeutics and our understanding of these disorders.

Oligopeptides arising from the degradation of self and non-self-proteins enter the antigen presentation pathway, activating humoral and cell-mediated immune responses against infected and cancerous cells. While peptides derived from exogenous proteins, acquired externally through the endo-lysosomal pathway are loaded on MHC-II molecules, peptides derived from endogenously synthesized proteins are presented on MHC-I (Vyas et al., 2008). Such peptides form the basis of CD8^+^ T-cell immune responses that play an important role in various infections, tumors, and autoimmune disorders. However, efficient presentation of endogenous foreign peptides requires them to compete with both high and low-affinity self-peptides originating from the cellular proteome. This competition would be puzzling in viral infections, where slow synthesis and degradation of viral proteins cannot explain the rapid onset of the adaptive immune response (Yewdell et al., 1996).

**FIGURE 1:**
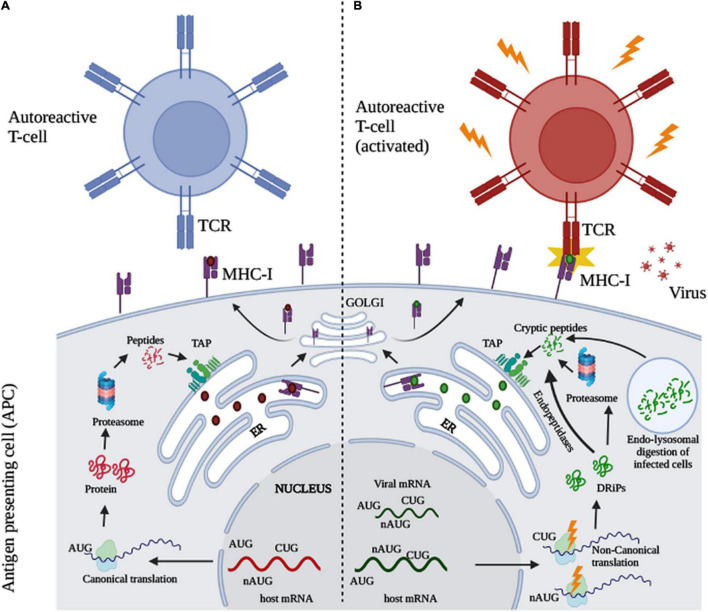
Role of non-canonical translation in virus-induced autoimmunity. (A) Proteins synthesized from canonical open reading frames (ORFs) are degraded *via* the proteasomal degradation pathway. The peptides produced thereof localize to the endoplasmic reticulum (ER) *via* TAP and are loaded on MHC-I molecules. The presented complexes however, fail to activate autoreactive T-cells as T cells against a majority of self-peptides are eliminated during the selection process in the thymus. (B) During viral infection, stress responses alter the translational machinery to synthesize novel proteins/polypeptides from both viral and cellular non-canonical ORFs, initiating at non-canonical AUG (nAUG) or other codons (e.g., CUG). These novel proteins are highly unstable and are preferentially degraded *via* several mechanisms including both proteasomal independent and dependent mechanisms, cytoplasmic endopeptidases and peptides formed through endo-lysosomal digestion of infected cells. The novel cryptic peptides, previously invisible to the immune system can now activate specific T-cell clones triggering an autoimmune-like response (Created using biorender.com).

In this context, the DRiPs (defective ribosomal products) hypothesis proposed by Yewdell and colleagues in 1996 describes a class of newly synthesized, highly unstable rapidly degraded polypeptides which forms the bulk of the antigenic repertoire in viral infections through their preferential presentation on MHC-I molecules (Yewdell et al., 1996; Table 1). Strong evidence in favor of DRiPs has explained immunosurveillance in infection and disease. A class of these products include peptides arising from non-canonical open reading frames (ORFs) (Yewdell et al., 2019)—translation initiation from alternative open reading frames (ARFs) (Bullock et al., 1997; Ronsin et al., 1999) at near-cognate start codons (e.g., CUG) (Schwab et al., 2004; Yang et al., 2016) or genomic regions previously deemed as untranslated (5′ or 3′ UTRs) (Laumont et al., 2016)—outside the annotated genome. High-throughput sequencing approaches combined with mass spectrometry have revealed that a substantial portion of our genome comprises of these non-canonical ORFs (Laumont et al., 2016; Erhard et al., 2018) and that their products are efficiently presented on MHC-I (Laumont et al., 2016; Erhard et al., 2018). Furthermore, the induction of cellular non-canonical translation is observed in cancers and viral infections (Laumont et al., 2016; Zanker et al., 2019; Chong et al., 2020). Given the immunological role of non-canonical gene products under infection and disease, we speculate that these peptides may play a role in autoimmunity, where they would be absent or weakly presented during T-cell selection, mediating their escape. Substantial synthesis, presentation, and processing of these peptides under infection, would then activate the circulating T-cells, triggering autoimmunity (Figure 1). To support our theory, we discuss the contribution of non-canonical translation products as MHC-I-associated peptides (MAPs), their significance in immunosurveillance and present evidence for their emerging role in autoimmunity in the context of viral infections, including COVID-19.

**TABLE 1:** Glossary of terminologies representing the nature of peptides in this study.

Canonical ORFs	ORFs encoding functional gene products/proteins exhibiting high stability and normal turnover kinetics in line with the conventional antigen processing and presentation pathway (Yewdell et al., 2019).
Non-canonical ORFs	ORFs ncoding highly unstable gene products, exhibiting distinct biological properties (Yewdell et al., 2019) including ORFs initiating at non-AUG start codons (Starck and Shastri, 2011) and may include unannotated ORFs comprising sORFs, uORFs, and ARFs (Erhard et al., 2018).
ARF (Alternative open reading frame)	ARFs comprise ORFs initiating within a gene body where such an ORF does not constitute the dominant canonical ORF representing the locus. Such initiation can be attributed to alternative transcription and translation start sites (Whisnant et al., 2020).
Annotated ORFs	ORFs currently annotated for a given organism.
Unannotated ORFs	ORFs identified from high-throughput sequencing experiments but currently not annotated.
uORF (upstream ORF)	Upstream ORFs are located upstream of a canonical ORF, which may function in regulating their downstream ORFs (Vattem and Wek, 2004) or provide a source of antigenic peptides (Starck and Shastri, 2016).
sORF (short ORF)	Short ORFs <100 aa in length, whose products would include unstable polypeptides which may perform biological functions or provide a source of antigenic peptides (Erhard et al., 2018).
Cryptic peptides	The term cryptic peptides include “hidden” or “invisible” peptides originating from genomic loci not previously annotated/studied (Erhard et al., 2020). Such peptides can be translated by both non-canonical and previously unannotated ORFs. In the context of autoimmunity, these peptides are “hidden” or “invisible” as described by Lanzavecchia (1995), due to their absence or low levels of expression which can hamper their antigen presentation, thereby explaining T-cells escaping self-tolerance against these peptides.
DRiPs (Defective ribosomal products)	The term DRiPs (Defective ribosomal products) coined by Yewdell et al. (1996), was used to describe a source of nascent polypeptides, highly unstable in nature exhibiting half-lives in the order of minutes and capable of producing MHC-I antigenic peptides. Such peptides are preferentially degraded due to their instability and biogenesis—including alterations in translation, ribosomal modifications and post-translational mechanisms including their rapid degradation and channeling (Yewdell et al., 2019).

## Generation of MHC-I Antigenic Peptides and Non-Canonical Translation

Most canonical viral and cellular proteins are stable and exhibit normal turnover kinetics prior to their degradation *via* both proteasomal dependent and independent mechanisms (Yewdell et al., 2019). The resulting peptides are transported into the endoplasmic reticulum (ER) *via* TAP (transporter associated with antigen processing) where their N-termini are cleaved *via* ER aminopeptidases (ERAPs), generating oligopeptides to be loaded on MHC-I (Starck and Shastri, 2011). Peptide-deficient MHC-I molecules are rendered unstable and fail to reach the cell surface, but undergo recycling to and from the cell surface upon successfully loading MHC-I peptides (Ljunggren et al., 1990). During infection and cancer, foreign peptides must compete with these self-peptides to mark infected or cancerous cells for CD8^+^ T-cell cytotoxicity. Besides, highly abundant herpesvirus proteins like EBNA (Dantuma et al., 2000) and LANA (Kwun et al., 2011) are extremely stable and less susceptible to immediate proteasomal degradation (van de Weijer et al., 2015). This makes it not only competitive, but also implausible for antigenic peptides to be presented in a timely manner (van de Weijer et al., 2015). A key feature of the antigen presentation pathway lies in selectively and preferentially presenting peptides on MHC molecules (Yewdell et al., 2019). This is achieved through rapidly degraded polypeptides derived from DRiPs and SLiPs (short-lived proteins) (Yewdell et al., 1996, 2019). The half-life of these proteins is in the order of minutes, ensuring rapid presentation of their peptides which is directly proportional to the translational rates of their source proteins. Many translational and post-translational mechanisms have explained the selectivity in presenting DRiPs (Yewdell et al., 2019). Accumulating evidence suggests that non-canonical translation is a prevalent phenomenon in mammalian cells, especially in infection and disease. These events include translation of loci outside the annotated genome, within 5′ and 3′ UTRs, at near-cognate start codons and include several alternative open reading frames (ARFs) and non-canonical ORFs resulting from premature stop-codon (PTC) read-through. Since these regions lie in previously unannotated or unknown regions, their peptide products are termed as cryptic peptides.

The concept of non-canonical translation emerged from the “pepton” hypothesis proposed by Boon and Van Pel (1989), where transfected genes devoid of distinct expression systems could produce polypeptides. Over the years, such translation events were observed for mammalian and viral genomes too. These non-canonical proteins gave rise to functional polypeptides as well as regulatory elements like upstream ORFs (uORFs) involved in translationally regulating their downstream ORFs (Vattem and Wek, 2004; Young and Wek, 2016). In fact, viruses could modulate non-canonical translation as seen in Vaccinia and Semliki Forest virus (SFV) (Townsend et al., 1988; Berglund et al., 2007), where insertion of genes into the respective viral vectors, led to the synthesis of novel polypeptides providing a rich source of DRiPs. Influenza DRiPs studies performed by Yewdell and colleagues laid the foundation to describe the role of non-canonical translation in antigen presentation. By cloning a Kb restricted model epitope, SIINFEKL, into IAV Neuraminidase (NA), they were able to distinguish non-canonical NA derived DRiPs products from the full-length NA protein. The translation of NA DRiP occurred independently of full-length NA mRNA initiating at CUG (Dolan et al., 2010) by specifically preventing nuclear export of NA mRNA, drastically reducing NA protein synthesis, without hampering levels of the NA DRiP product. Indeed, non-canonical translation is governed by several factors, including differential usage of ribosomal proteins, which strictly control the global translation and MHC-I presentation of non-AUG and ARF derived polypeptides (Wei et al., 2019). These studies highlight key features of non-canonical proteins by shedding light on the differences in their processing pathways to provide a wealth of antigenic peptides.

## Immunological Significance of Non-Canonical Translation and Defective Ribosomal Products

The contribution of cryptic peptides in antigen presentation emerged from pioneering studies by Shastri and colleagues. They demonstrated an antigenic peptide whose source protein initiated at a non-canonical codon, CUG, translating a leucine instead of a methionine, and could activate specific alloreactive T cell clones (Schwab et al., 2004). Decoding CUG was a result of translational control mediated by eIF2A phosphorylation under cellular stress and was not affected by compounds affecting initiation at AUG (Schwab et al., 2004). Phosphorylation of eIF2A is a hallmark of cellular stress, often seen in viral infections and plays a role in the translational regulation of upstream ORFs (uORFs) (Vattem and Wek, 2004; Young and Wek, 2016). These uORFs also form a source of cryptic peptides as observed in the 5′ UTR cryptic product of the VEGF gene in renal cell carcinoma (Weinzierl et al., 2008). It was later shown that translation initiation at CUG could be enhanced *via* inflammatory stimuli and viral infections by modulating the ribosomal machinery (Prasad et al., 2016). More recently, the contribution of interferon in rapidly synthesizing and presenting DRiPs was observed in the context of MHC associated peptides (Komov et al., 2021).

Viruses have adopted several means of translational control. Non-canonical translation has been reported for Plautia stali intestinal virus through internal ribosome entry site (IRES) dependent translation of the capsid protein, initiating at a non-canonical CAA codon (Sasaki and Nakashima, 2000). Non-canonical translation also forms a source of antigenic epitopes as observed in the case of Influenza (IAV) DRiPs synthesis (Dolan et al., 2010; Yang et al., 2016), portraying their immunological role in viral infections. Generation of these anti-viral cryptic epitopes through non-canonical translation has been well documented and reviewed in retroviruses (Starck and Shastri, 2011) where ribosomal frameshifting and ARFs have contributed to non-conventional epitopes derived from gag, pol and env genes in murine AIDS, HIV and Simian Immunodeficiency Virus (SIV) (Mayrand et al., 1998; Cardinaud et al., 2004; Ho and Green, 2006). Protective CD8^+^ T-cell responses were elicited in these cases, especially in SIV infection of rhesus macaques, where a substantial portion of T-cell responses were attributed to cryptic peptides arising from ARFs (Maness et al., 2010). The fact that ARF derived epitopes appeared to be more immunologically dominant than classical epitopes upon assaying PBMCs against both ARF and non-ARF epitopes (Maness et al., 2010) warrant the use of such peptides in therapy to trigger cytotoxic CD8^+^ T cells. In case of HIV, acquired mutations in ARF derived epitopes prevented their presentation, stressing on the importance of these epitopes in immunosurveillance and how viruses could evolve to antagonize immune responses (Maness et al., 2007; Berger et al., 2010). Significant improvements in understanding the plethora of gene products encoded by genomes came from studies employing ribosome profiling, a technique applied to study translation events by sequencing ribosome protected RNA (Ingolia et al., 2012). Ribosome profiling revealed hundreds of novel open reading frames in several viruses and cancers, severely undermining our knowledge of the coding capacity of mammalian and viral genomes (Stern-Ginossar et al., 2012; Arias et al., 2014; Chong et al., 2020; Whisnant et al., 2020; Ruiz Cuevas et al., 2021). These novel ORFs comprised of many short open reading frames (sORFs), which formed the major component of the non-canonical “ORFeome.” Such sORFs resemble DRiPs products in that the encoded microproteins commonly initiate at near-cognate start codons, appear to be highly unstable and disordered in structure (Ruiz Cuevas et al., 2021). The instability of sORFs makes it challenging to detect them through conventional proteomic methods including shot gun mass spectrometry (MS) (Erhard et al., 2018). However, sORF-derived peptides (SEPs), like DRiPs, would efficiently enter the antigen presentation pathway. Indeed, such peptides were better recovered by MHC-I peptidomics due to the ability of MHC-I molecules to form stable complexes with antigenic peptides and like DRiPs, the extent of their presentation correlated with their translational rates (Erhard et al., 2018). MHC-I-peptidomics revealed that approximately 10% of the MHC-I-immunopeptidome comprised of cryptic peptides originating from non-canonical ORFs (Erhard et al., 2018; Ruiz Cuevas et al., 2021). Immunopeptidomic studies (Erhard et al., 2020) and proteogenomic approaches utilizing MS coupled with high-throughput sequencing improved the identification of novel neo-antigens in tumors supporting the role of non-canonical peptides in immunity which were efficiently presented on MHC-I molecules (Laumont et al., 2016; Chong et al., 2020; Ouspenskaia et al., 2021).

## Non-Canonical Translation and Defective Ribosomal Products in Autoimmunity

T-cell lymphocytes expressing either CD4^+^ or CD8^+^ TCRs are capable of recognizing antigen associated MHC class II and I molecules respectively (Kumar et al., 2018). During their development in the thymus, T-cells undergo positive and negative selection, where T-cells recognizing self-antigen MHC complexes with low to moderate affinity undergo proliferation, whereas strong interactions with high affinity self-antigen MHC complexes leads to cell death, preventing autoimmunity, while some of the cells differentiate into regulatory T-cells (Takaba and Takayanagi, 2017). The self-tolerant T-cells selected recognize abnormal or foreign peptides during infection and disease. However, some T-cells escape tolerance and are activated when exposed to self-peptides which may not have been expressed during their development at optimal levels. These “invisible” peptides are therefore cryptic in nature and may not be sufficiently expressed during T-cell selection (Sercarz et al., 1993; Lanzavecchia, 1995). In certain cases, cryptic epitopes would have lower affinity for MHC molecules and may not be presented due to competition with the high affinity epitopes. However, inflammatory stimuli and co-stimulatory signals might alter their expression and processing, thereby activating self-reactive T-cells.

Evidence for T-cells reactive toward self-cryptic epitopes has been extensively reviewed for a variety of autoimmune disorders (Warnock and Goodacre, 1997). Studies conducted for rhesus polypeptides involved in autoimmune hemolytic anemia (AIHA) analyzed T-cell responses toward the full-length autoantigen and synthetic peptides derived from the same (Barker and Elson, 1994). The study described *in vitro* experiments with intact protein which failed to elicit self-specific T-cell responses as opposed to peptide constructs, indicating that the latter comprised of a pool of cryptic peptides for which T-cells had escaped self-tolerance *in vivo*, and that these peptides were not synthesized *in vivo*, explaining their cryptic nature. The fact that full-length proteins could not trigger such responses implied that their processing pathways did not efficiently present cryptic epitopes to activate the T-cells. A similar finding was reported for proteolytic protein (PLP) involved in multiple sclerosis (MS) (Markovic-Plese et al., 1995) and thyroid peroxidase (TPO) involved in Grave’s disease (Quaratino et al., 1996) where the antigenic peptides significantly differed for both endogenously and exogenously processed TPO proteins. These features of cryptic autoantigens bear close resemblance to DRiPs and non-canonical translation products. Cartilage autoantigens in Rheumatoid arthritis (RA) (Buttle et al., 1995) rapidly degrading to give rise to antigenic peptides draws similarities to the unstable nature of DRiPs, explaining their preferential presentation during disease. Epitope processing is also regulated by changes in the activity of cytosolic endopeptidases that play an important role in exposing cryptic epitopes within the myelin basic protein (MBD) (Anderton et al., 2002). Whether these peptides originate from non-canonical translation events remains an open question since most of the studies exploited synthetic peptide constructs, making it difficult to comment on the nature and generation of their source proteins, compounded by the lack of studies in this direction.

Recent studies provide a clearer picture to explain the involvement of non-canonical translation in autoimmune disorders. CD8^+^ T-cell isolated from Reiter’s syndrome patients led to the identification of an autoreactive T cells specific toward an epitope encoded by human Interleukin-10 (IL-10) (Saulquin et al., 2002). T-cells were activated upon transfecting the nucleotide sequence encoding the epitope but failed to generate immune responses when utilizing synthetic peptides. It was later found by mutational analysis, that this epitope was formed because of non-canonical frameshifting, resulting in a peptide encoded by both ORF1 and ORF2. Given the immunosuppressive role of IL-10, the deletion of IL-10 producing cells could have a profound impact on the pathology of Reiter’s syndrome. The potential for non-canonical self-peptides to be immunologically relevant has also been observed in therapeutics. Defective proteins synthesized due to a premature stop codon (PTC) form the basis of diseases like cystic fibrosis and Duchenne muscular dystrophy (Goodenough et al., 2014). The use of aminoglycosides has the potential to cause read-through leading to the synthesis of full-length proteins to overcome this. However, stop codon read-through opens possibilities of non-canonical translation events downstream, which has been observed in gentamicin treated cells leading to the development of immunologically relevant self-peptides.

A breakthrough in understanding the contribution of non-canonical translation products or DRiPs to autoimmunity comes from recent studies in Type I diabetes (T1D) (Kracht et al., 2017). In their elegant study, Kracht and colleagues report a novel DRiP encoded by Insulin mRNA (INS-DRiP) in the non-canonical +2 open reading frame initiating at an AUG start codon. The cryptic epitope encoded by INS-DRiPs efficiently entered the antigen presentation pathway and was surprisingly loaded on both HLA I and II molecules. Such a response would generate both CD8^+^ T-cell cytotoxic responses and autoantibodies through CD4^+^ helper T-cells.

## Can Viruses Induce Autoimmunity Through Non-Canonical Peptides and Defective Ribosomal Products?—A Theory

Viruses have shown to contribute to the pathophysiology of several autoimmune disorders through a broad range of mechanisms (Smatti et al., 2019). They can encode for cross-reactive structural or molecular “mimics” of several autoantigens that can activate self-specific T-cells and autoantibodies—a concept defined as molecular mimicry. On the other hand, certain viruses induce autoimmunity through bystander activation of T-cells and antigen presenting cells (APCs) as well as epitope spreading. These mechanisms rely on the exposure of self-peptides to autoreactive T-cells through destruction of host cells stimulated by APCs and a pro-inflammatory microenvironment. Several viruses including IAV, Coxsackie viruses and herpesviruses including HCMV, EBV, HSV-1, and HHV-6A mediate self-reactivity through these processes (Smatti et al., 2019). While the mechanisms modulate the clinical symptoms of autoimmune disorders, they do not provide adequate information about how self-reactive cells were generated at the first place nor provide information about the synthesis and processing of autoantigenic peptides. Thymic T-cells must have escaped tolerance due to poor availability of certain peptides which remain “hidden” or are cryptic and viral infections may contribute in generating such peptides through non-canonical synthesis or processing of their source proteins (Sercarz et al., 1993; Lanzavecchia, 1995).

Indeed, evidence to define the role of cryptic peptides in virus-induced autoimmunity emerged from studies in HIV infections where the viral membrane protein, gp120 was able to modulate the processing of endogenous CD4^+^ to generate self-peptides (Salemi et al., 1995). Expression of gp120 led to an increase in CD4^+^ endocytic processing, generating novel epitopes which in turn generated anti-CD4 autoantibodies. Synthesis and presentation of less immunodominant and normally absent epitopes was also observed in the case of Theiler’s virus mediated MS (Miller et al., 1997). Differences in the processing and magnitude of antigen presentation between non-canonical and canonical peptides must play a crucial role. In the case of HSV-1-induced HSK, peptides originating from the viral protein, UL6 appeared to be cross-reactive when exposed to corneal T-cell clones in murine models (Zhao et al., 1998; Smatti et al., 2019). However, HSK studies in patients did not result in the isolation of T cells cross-reactive with UL6 indicating other mechanisms at play, which may involve self-antigens expressed at sufficiently high levels to trigger autoimmune T-cells (Verjans et al., 2000). Similarly, for EBV, anti-Ro antibodies found in SLE patients were cross-reactive with the EBV EBNA-1 protein (Poole et al., 2006). Synthetic peptides derived from Ro generated a broad range of antibodies against several epitopes including autoantibodies in mice, while immunization with intact Ro protein only produced antibodies against the immunizing peptide indicating differences in peptide processing during viral infections. While epitope spreading and mimicry explain the development of autoimmunity in viral infections, mechanistic differences in the generation of autoantigens must be addressed.

The most profound example for virus induced autoimmunity in the context of non-canonical translation comes from studies in IAV infection, where generation of viral DRiPs resulting from non-canonical translation through an ARF also results in the generation of a cellular ARF DRiP product (Zanker et al., 2019). The study described for the first time that viruses can induce cellular DRiPs, which has implications in understanding virus-induced autoimmunity. The authors conclude by stating that viruses might trigger autoimmunity by simply generating DRiPs. This might explain the preferential presentation of autoantigens under infection which generally remain hidden from developing T-cells. Perhaps a large amount of DRiPs derived peptides originate from non-canonical ORFs and remain invisible to the immune system under normal conditions that do not permit expression of such ORFs. Ribosome profiling conducted in human Beta cells in both normal and type 1 diabetes (T1D) conditions (Thomaidou et al., 2021) have revealed a novel set of non-canonical polypeptides which might unveil neo-antigens in T1D. Future studies to understand the role of novel ORFs in autoimmunity might explain several non-canonical autoantigens. Indeed, viruses encode many non-canonical ORFs that may play a role in better understanding virus-induced autoimmunity (Stern-Ginossar et al., 2012; Arias et al., 2014; Whisnant et al., 2020).

## A Perspective on SARS-CoV2 Induced Autoimmunity—Can Cryptic Peptides Play a Role?

Recent evidence has indicated that severe acute respiratory syndrome coronavirus 2 (SARS-CoV2) affects the outcome of autoimmune disorders. The virus responsible for the COVID-19 pandemic has been associated with the development of pediatric inflammatory multisystemic syndrome (PIMS) or Kawasaki-like disease in children as well as Guillain-Barre syndrome (GBS) and Miller Fisher syndrome (MFS) (Dotan et al., 2021). Clinical studies establishing an association between SLE and SARS-CoV2 disease firmly establish a link between the two (Gracia-Ramos and Saavedra-Salinas, 2021; Sjöwall et al., 2021; Zamani et al., 2021). As observed in other viruses, the mechanisms triggering autoimmunity must be similar for SARS-CoV2. *In silico* studies have predicted 23 peptides shared by the virus and the human proteome present in the canonical ORFs of SARS-CoV2 (Karami Fath et al., 2021), of which three were experimentally confirmed to bind to HLA. However, the significance of these peptides to function as cross-reactive mimics is yet to be confirmed.

**FIGURE 2:**
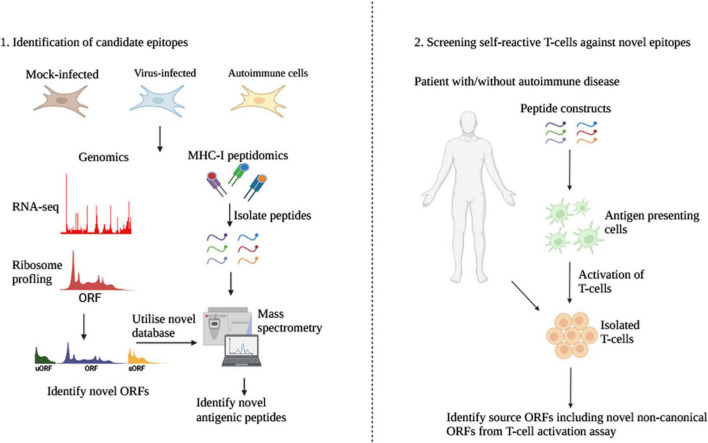
Assessing the role of non-canonical viral ORFs in virus-induced autoimmunity. To support our presented hypothesis, a proteogenomic approach should be adopted to identify novel ORFs combining RNA-seq and ribosome profiling methods for the displayed conditions (1). The novel database would then be utilized to identify novel antigenic epitopes presented by MHC-I complexes through solubilization of peptides stably bound to MHC-I, which would significantly improve the determination of highly unstable non-canonical ORFs of lengths <100 aa, which would otherwise be challenging to detect using conventional proteomics. The identified peptides should then be utilized for uptake by antigen presenting cells *in vitro* to assess self-reactivity of T-cells isolated from patients with and without autoimmune diseases and with a history of chronic viral infection for the virus under study (2). This methodology would help determine both the proportion and immunological repertoire of non-canonical peptides playing a role in virus-induced autoimmunity. By deciphering their expression under various conditions and cell lines (1), one could determine both the non-canonical nature and expression of ORFs and if indeed their absence or weak expression could explain an escape in immunological self-tolerance for the specific peptide and its source protein. sORF, short ORF; uORF, upstream ORF; APCs, antigen presenting cells (Created using biorender.com).

SARS-CoV2 utilizes ACE-2 receptors for virus entry (Jackson et al., 2021). ACE-2 is highly expressed in patients with COVID-19. A theory proposing the role of soluble ACE-2 in autoimmunity posits that ACE-2 molecules circulating in plasma would be endocytosed by APCs to produce novel antigens that can trigger autoantibodies (McMillan et al., 2021). The synthesis of such novel peptides can occur due to alternative processing of soluble ACE-2 in the endo-lysosomal compartment or the engulfment of non-canonical ACE-2 polypeptides. At the translational level, peptides might be generated from cellular proteins by non-canonical translation. Certainly, autoantibodies against cellular proteins including anti-Ro have been observed in patients affected by COVID-19 (Fujii et al., 2020). Differences in the variety of non-canonical epitopes arising from Ro protein and transfected Ro nucleotide sequence is a clear example of non-canonical translational regulation (Poole et al., 2006). The induction of several cytokines like IL-6 and a strong pro-inflammatory response is a classical feature of COVID-19 which may promote cryptic translation due to stress-mediated alteration of the translational machinery (Chen et al., 2020). While the contribution of non-canonical translation in SARS-CoV2 induced autoimmunity remains speculative, ribosome profiling has revealed cryptic products encoded by SARS-CoV2 (Finkel et al., 2021), expanding our view of the viruses’ coding capacity. Since epitopes are predicted using a proteomic database to screen for antigenic peptides, non-canonical proteins, particularly unstable and difficult to detect *via* proteomics, are neglected without knowledge of their ORF sequence. ORF prediction through ribosome profiling coupled with MHC-I peptidomics will hence improve epitope detection and validation for various diseases (Erhard et al., 2018) to better understand the role of non-canonical gene products in autoimmunity (Figure 2). Indeed, some of the cryptic products encoded by SARS-CoV2 were confirmed through proteomics (Schmidt et al., 2021).

## Discussion

We provide a perspective on how non-canonical translation and generation of cryptic epitopes can improve our understanding of autoimmune disorders in the context of viral infections. The theory discussed would help explain how T cells escape self-tolerance and how cryptic antigens might trigger them. Non-canonical expression of cryptic peptides due to infection and cellular stress will explain why they remain hidden from T cells under normal conditions. Although direct evidence for the contribution of non-canonical translation remains elusive, ribosome profiling data coupled with mass spectrometry has highlighted the breadth of cryptic gene products in mammalian and viral genomes, laying the foundation for their functional characterization in the context of immunosurveillance. Autoantigen-ome studies in A549 lung cells (Wang et al., 2021) have revealed abnormal levels of around 291 altered autoantigens in SARS-CoV2 infection, out of which only 191 autoantigens are known to promote self-reactivity. These autoantigens could explain post infection sequelae persistent after acute COVD-19 disease in several patients, termed as “Long COVID”- whose symptoms and abnormalities share close resemblance to myalgic encephalomyelitis/chronic fatigue syndrome (ME/CFS) which is often associated with viral infections (Mackay, 2021). The fact that more than a third of the autoantigens remain unidentified in the context of autoimmunity, raise the possibility of cryptic peptides playing a role in “Long COVID” disease. Perhaps modulation of the cellular proteome by SARS-CoV2 infection would govern the atlas of putative self-peptides involved in autoimmunity.

## Data Availability Statement

The original contributions presented in the study are included in the article, further inquiries can be directed to the corresponding author.

## Author Contributions

ML and BKP conceived the idea. ML, FE, LD, and BKP wrote the manuscript. All authors contributed to the article and approved the submitted version.

## Conflict of Interest

The authors declare that the research was conducted in the absence of any commercial or financial relationships that could be construed as a potential conflict of interest.

## Publisher’s Note

All claims expressed in this article are solely those of the authors and do not necessarily represent those of their affiliated organizations, or those of the publisher, the editors and the reviewers. Any product that may be evaluated in this article, or claim that may be made by its manufacturer, is not guaranteed or endorsed by the publisher.

## References

[B1] Anderton S. M. Viner N. J. Matharu P. Lowrey P. A. Wraith D. C. (2002). Influence of a dominant cryptic epitope on autoimmune T cell tolerance. *Nat. Immunol.* 3 175–181. 10.1038/ni756 11812995

[B2] Arias C. Weisburd B. Stern-Ginossar N. Mercier A. Madrid A. S. Bellare P. (2014). KSHV 2.0: a comprehensive annotation of the Kaposi’s sarcoma-associated *herpesvirus* genome using next-generation sequencing reveals novel genomic and functional features. *PLoS Pathog.* 10:e1003847. 10.1371/journal.ppat.1003847 24453964 PMC3894221

[B3] Barker R. N. Elson C. J. (1994). Multiple self epitopes on the rhesus polypeptides stimulate immunologically ignorant human T cells in vitro. *Eur. J. Immunol.* 24 1578–1582. 10.1002/eji.1830240719 7517875

[B4] Berger C. T. Carlson J. M. Brumme C. J. Hartman K. L. Brumme Z. L. Henry L. M. (2010). Viral adaptation to immune selection pressure by HLA class I-restricted CTL responses targeting epitopes in HIV frameshift sequences. *J. Exp. Med.* 207 61–75. 10.1084/jem.20091808 20065065 PMC2812535

[B5] Berglund P. Finzi D. Bennink J. R. Yewdell J. W. (2007). Viral alteration of cellular translational machinery increases defective ribosomal products. *J. Virol.* 81 7220–7229. 10.1128/JVI.00137-07 17459927 PMC1933321

[B6] Boon T. Van Pel A. (1989). T cell-recognized antigenic peptides derived from the cellular genome are not protein degradation products but can be generated directly by transcription and translation of short subgenic regions. A hypothesis. *Immunogenetics* 29 75–79. 10.1007/BF00395854 2783681

[B7] Bullock T. N. Patterson A. E. Franlin L. L. Notidis E. Eisenlohr L. C. (1997). Initiation codon scanthrough versus termination codon readthrough demonstrates strong potential for major histocompatibility complex class I-restricted cryptic epitope expression. *J. Exp. Med.* 186 1051–1058. 10.1084/jem.186.7.1051 9314554 PMC2199058

[B8] Buttle D. J. Bramwell H. Hollander A. P. (1995). Proteolytic mechanisms of cartilage breakdown: a target for arthritis therapy? *Clin. Mol. Pathol.* 48 M167–M177. 10.1136/mp.48.4.m167 16696000 PMC407956

[B9] Cardinaud S. Moris A. Février M. Rohrlich P. S. Weiss L. Langlade-Demoyen P. (2004). Identification of cryptic MHC I-restricted epitopes encoded by HIV-1 alternative reading frames. *J. Exp. Med.* 199 1053–1063. 10.1084/jem.20031869 15078897 PMC2211898

[B10] Chen L. Y. C. Hoiland R. L. Stukas S. Wellington C. L. Sekhon M. S. (2020). Confronting the controversy: interleukin-6 and the COVID-19 cytokine storm syndrome. *Eur. Respir. J.* 56:2003006. 10.1183/13993003.03006-2020 32883678 PMC7474149

[B11] Chong C. Müller M. Pak H. Harnett D. Huber F. Grun D. (2020). Integrated proteogenomic deep sequencing and analytics accurately identify non-canonical peptides in tumor immunopeptidomes. *Nat. Commun.* 11:1293. 10.1038/s41467-020-14968-9 32157095 PMC7064602

[B12] Dantuma N. P. Heessen S. Lindsten K. Jellne M. Masucci M. G. (2000). Inhibition of proteasomal degradation by the gly-Ala repeat of Epstein-Barr virus is influenced by the length of the repeat and the strength of the degradation signal. *Proc. Natl. Acad. Sci. U.S.A.* 97 8381–8385. 10.1073/pnas.140217397 10890896 PMC26956

[B13] Dolan B. P. Knowlton J. J. David A. Bennink J. R. Yewdell J. W. (2010). RNA polymerase II inhibitors dissociate antigenic peptide generation from normal viral protein synthesis: a role for nuclear translation in defective ribosomal product synthesis? *J. Immunol.* 185 6728–6733. 10.4049/jimmunol.1002543 21048111 PMC3398797

[B14] Dotan A. Muller S. Kanduc D. David P. Halpert G. Shoenfeld Y. (2021). The SARS-CoV-2 as an instrumental trigger of autoimmunity. *Autoimmun. Rev.* 20:102792. 10.1016/j.autrev.2021.102792 33610751 PMC7892316

[B15] Erhard F. Dölken L. Schilling B. Schlosser A. (2020). Identification of the cryptic HLA-I immunopeptidome. *Cancer Immunol. Res.* 8 1018–1026. 10.1158/2326-6066.CIR-19-0886 32561536

[B16] Erhard F. Halenius A. Zimmermann C. L’Hernault A. Kowalewski D. J. Weekes M. P. (2018). Improved Ribo-seq enables identification of cryptic translation events. *Nat. Methods* 15 363–366. 10.1038/nmeth.4631 29529017 PMC6152898

[B17] Finkel Y. Mizrahi O. Nachshon A. Weingarten-Gabbay S. Morgenstern D. Yahalom-Ronen Y. (2021). The coding capacity of SARS-CoV-2. *Nature* 589 125–130. 10.1038/s41586-020-2739-1 32906143

[B18] Fujii H. Tsuji T. Yuba T. Tanaka S. Suga Y. Matsuyama A. (2020). High levels of anti-SSA/Ro antibodies in COVID-19 patients with severe respiratory failure: a case-based review : high levels of anti-SSA/Ro antibodies in COVID-19. *Clin. Rheumatol.* 39 3171–3175. 10.1007/s10067-020-05359-y 32844364 PMC7447083

[B19] Goodenough E. Robinson T. M. Zook M. B. Flanigan K. M. Atkins J. F. Howard M. T. (2014). ”Cryptic MHC class I-binding peptides are revealed by aminoglycoside-induced stop codon read-through into the 3′. UTR. *Proc. Natl. Acad. Sci. U.S.A.* 111 5670–5675. 10.1073/pnas.1402670111 24706797 PMC3992684

[B20] Gracia-Ramos A. E. Saavedra-Salinas M. (2021). Can the SARS-CoV-2 infection trigger systemic lupus erythematosus? A case-based review. *Rheumatol. Int.* 41 799–809. 10.1007/s00296-021-04794-7 33543338 PMC7861004

[B21] Ho O. Green W. R. (2006). Cytolytic CD8+ T cells directed against a cryptic epitope derived from a retroviral alternative reading frame confer disease protection. *J. Immunol.* 176 2470–2475. 10.4049/jimmunol.176.4.2470 16456007

[B22] Ingolia N. T. Brar G. A. Rouskin S. McGeachy A. M. Weissman J. S. (2012). The ribosome profiling strategy for monitoring translation in vivo by deep sequencing of ribosome-protected mRNA fragments. *Nat. Protoc.* 7 1534–1550. 10.1038/nprot.2012.086 22836135 PMC3535016

[B23] Jackson C. B. Farzan M. Chen B. Choe H. (2021). Mechanisms of SARS-CoV-2 entry into cells. *Nat. Rev. Mol. Cell Biol.* 23 3–20. 34611326 10.1038/s41580-021-00418-x PMC8491763

[B24] Karami Fath M. Jahangiri A. Ganji M. Sefid F. Payandeh Z. Hashemi Z. S. (2021). SARS-CoV-2 proteome harbors peptides which are able to trigger autoimmunity responses: implications for infection, vaccination, and population coverage. *Front. Immunol.* 12:705772. 10.3389/fimmu.2021.705772 34447375 PMC8383889

[B25] Komov L. Melamed Kadosh D. Barnea E. Admon A. (2021). The Effect of Interferons on Presentation of Defective Ribosomal Products as HLA Peptides. *Mol. Cell Proteomics* 20:100105. 10.1016/j.mcpro.2021.100105 34087483 PMC8724922

[B26] Kracht M. J. van Lummel M. Nikolic T. Joosten A. M. Laban S. van der Slik A. R. (2017). Autoimmunity against a defective ribosomal insulin gene product in type 1 diabetes. *Nat. Med.* 23 501–507. 10.1038/nm.4289 28263308

[B27] Kumar B. V. Connors T. J. Farber D. L. (2018). Human T cell development, localization, and function throughout life. *Immunity* 48 202–213. 10.1016/j.immuni.2018.01.007 29466753 PMC5826622

[B28] Kwun H. J. da Silva S. R. Qin H. Ferris R. L. Tan R. Chang Y. (2011). The central repeat domain 1 of Kaposi’s sarcoma-associated herpesvirus (KSHV) latency associated-nuclear antigen 1 (LANA1) prevents cis MHC class I peptide presentation. *Virology* 412 357–365. 10.1016/j.virol.2011.01.026 21324504 PMC3097433

[B29] Lanzavecchia A. (1995). How can cryptic epitopes trigger autoimmunity? *J. Exp. Med.* 181 1945–1948. 10.1084/jem.181.6.1945 7539032 PMC2192044

[B30] Laumont C. M. Daouda T. Laverdure J. P. Bonneil E. Caron-Lizotte O. Hardy M. P. (2016). Global proteogenomic analysis of human MHC class I-associated peptides derived from non-canonical reading frames. *Nat. Commun.* 7:10238. 10.1038/ncomms10238 26728094 PMC4728431

[B31] Ljunggren H. G. Stam N. J. Ohlén C. Neefjes J. J. Höglund P. Heemels M. T. (1990). Empty MHC class I molecules come out in the cold. *Nature* 346 476–480. 10.1038/346476a0 2198471

[B32] Mackay A. (2021). A paradigm for post-covid-19 fatigue syndrome analogous to ME/CFS. *Front. Neurol.* 12:701419. 10.3389/fneur.2021.701419 34408721 PMC8365156

[B33] Maness N. J. Valentine L. E. May G. E. Reed J. Piaskowski S. M. Soma T. (2007). AIDS virus specific CD8+ T lymphocytes against an immunodominant cryptic epitope select for viral escape. *J. Exp. Med.* 204 2505–2512. 10.1084/jem.20071261 17954573 PMC2118485

[B34] Maness N. J. Walsh A. D. Piaskowski S. M. Furlott J. Kolar H. L. Bean A. T. (2010). CD8+ T cell recognition of cryptic epitopes is a ubiquitous feature of AIDS virus infection. *J. Virol.* 84 11569–11574. 10.1128/JVI.01419-10 20739530 PMC2953171

[B35] Markovic-Plese S. Fukaura H. Zhang J. Al-Sabbagh A. Southwood S. Sette A. (1995). T cell recognition of immunodominant and cryptic proteolipid protein epitopes in humans. *J. Immunol.* 155 982–992. 7541828

[B36] Mayrand S. M. Schwarz D. A. Green W. R. (1998). An alternative translational reading frame encodes an immunodominant retroviral CTL determinant expressed by an immunodeficiency-causing retrovirus. *J. Immunol.* 160 39–50. 9551954

[B37] McMillan P. Dexhiemer T. Neubig R. R. Uhal B. D. (2021). COVID-19-A theory of autoimmunity against ACE-2 explained. *Front. Immunol.* 12:582166. 10.3389/fimmu.2021.582166 33833750 PMC8021777

[B38] Miller S. D. Vanderlugt C. L. Begolka W. S. Pao W. Yauch R. L. Neville K. L. (1997). Persistent infection with Theiler’s virus leads to CNS autoimmunity via epitope spreading. *Nat. Med.* 3 1133–1136. 10.1038/nm1097-1133 9334726

[B39] Ouspenskaia T. Law T. Clauser K. R. Klaeger S. Sarkizova S. Aguet F. (2021). Unannotated proteins expand the MHC-I-restricted immunopeptidome in cancer. *Nat. Biotechnol.* 10.1038/s41587-021-01021-3 [Epub ahead of print]. PMC10198624 34663921

[B40] Poole B. D. Scofield R. H. Harley J. B. James J. A. (2006). Epstein-Barr virus and molecular mimicry in systemic lupus erythematosus. *Autoimmunity* 39 63–70. 10.1080/08916930500484849 16455583

[B41] Prasad S. Starck S. R. Shastri N. (2016). Presentation of cryptic peptides by MHC class I is enhanced by inflammatory stimuli. *J. Immunol.* 197 2981–2991. 10.4049/jimmunol.1502045 27647836 PMC5101130

[B42] Quaratino S. Feldmann M. Dayan C. M. Acuto O. Londei M. (1996). Human self-reactive T cell clones expressing identical T cell receptor beta chains differ in their ability to recognize a cryptic self-epitope. *J. Exp. Med.* 183 349–358. 10.1084/jem.183.2.349 8627148 PMC2192455

[B43] Ronsin C. Chung-Scott V. Poullion I. Aknouche N. Gaudin C. Triebel F. (1999). A non-AUG-defined alternative open reading frame of the intestinal carboxyl esterase mRNA generates an epitope recognized by renal cell carcinoma-reactive tumor-infiltrating lymphocytes in situ. *J. Immunol.* 163 483–490. 10384152

[B44] Ruiz Cuevas M. V. Hardy M. P. Holly J. Bonneil E. Durette C. Courcelles M. (2021). Most non-canonical proteins uniquely populate the proteome or immunopeptidome. *Cell Rep.* 34:108815. 10.1016/j.celrep.2021.108815 33691108 PMC8040094

[B45] Salemi S. Caporossi A. P. Boffa L. Longobardi M. G. Barnaba V. (1995). HIVgp120 activates autoreactive CD4-specific T cell responses by unveiling of hidden CD4 peptides during processing. *J. Exp. Med.* 181 2253–2257. 10.1084/jem.181.6.2253 7760011 PMC2192056

[B46] Sasaki J. Nakashima N. (2000). Methionine-independent initiation of translation in the capsid protein of an insect RNA virus. *Proc. Natl. Acad. Sci. U.S.A.* 97 1512–1515. 10.1073/pnas.010426997 10660678 PMC26465

[B47] Saulquin X. Scotet E. Trautmann L. Peyrat M. A. Halary F. Bonneville M. (2002). +1 Frameshifting as a novel mechanism to generate a cryptic cytotoxic T lymphocyte epitope derived from human interleukin 10. *J. Exp. Med.* 195 353–358. 10.1084/jem.20011399 11828010 PMC2193594

[B48] Schmidt N. Lareau C. A. Keshishian H. Ganskih S. Schneider C. Hennig T. (2021). The SARS-CoV-2 RNA-protein interactome in infected human cells. *Nat. Microbiol.* 6 339–353. 10.1038/s41564-020-00846-z 33349665 PMC7906908

[B49] Schwab S. R. Shugart J. A. Horng T. Malarkannan S. Shastri N. (2004). Unanticipated antigens: translation initiation at CUG with leucine. *PLoS Biol.* 2:e366. 10.1371/journal.pbio.0020366 15510226 PMC524250

[B50] Sercarz E. E. Lehmann P. V. Ametani A. Benichou G. Miller A. Moudgil K. (1993). Dominance and crypticity of T cell antigenic determinants. *Annu. Rev. Immunol.* 11 729–766. 10.1146/annurev.iy.11.040193.003501 7682817

[B51] Sjöwall J. Azharuddin M. Frodlund M. Zhang Y. Sandner L. Dahle C. (2021). SARS-CoV-2 antibody isotypes in systemic lupus erythematosus patients prior to vaccination: associations with disease activity, antinuclear antibodies, and immunomodulatory drugs during the first year of the pandemic. *Front. Immunol.* 12:724047. 10.3389/fimmu.2021.724047 34512651 PMC8430325

[B52] Smatti M. K. Cyprian F. S. Nasrallah G. K. Al Thani A. A. Almishal R. O. Yassine H. M. (2019). Viruses and autoimmunity: a review on the potential interaction and molecular mechanisms. *Viruses* 11:762. 10.3390/v11080762 31430946 PMC6723519

[B53] Starck S. R. Shastri N. (2011). Non-conventional sources of peptides presented by MHC class I. *Cell. Mol. Life Sci.* 68 1471–1479. 10.1007/s00018-011-0655-0 21390547 PMC3071930

[B54] Starck S. R. Shastri N. (2016). Nowhere to hide: unconventional translation yields cryptic peptides for immune surveillance. *Immunol. Rev.* 272 8–16. 10.1111/imr.12434 27319338 PMC4916849

[B55] Stern-Ginossar N. Weisburd B. Michalski A. Le V. T. Hein M. Y. Huang S. X. (2012). Decoding human cytomegalovirus. *Science* 338 1088–1093. 23180859 10.1126/science.1227919 PMC3817102

[B56] Takaba H. Takayanagi H. (2017). The mechanisms of T cell selection in the thymus. *Trends Immunol.* 38 805–816. 28830733 10.1016/j.it.2017.07.010

[B57] Thomaidou S. Slieker R. C. van der A. R. Slik J. Boom Mulder F. Munoz-Garcia A. (2021). Long RNA sequencing and ribosome profiling of inflamed β-cells reveal an extensive translatome landscape. *Diabetes* 70 2299–2312. 10.2337/db20-1122 34554924

[B58] Townsend A. Bastin J. Gould K. Brownlee G. Andrew M. Coupar B. (1988). Defective presentation to class I-restricted cytotoxic T lymphocytes in vaccinia-infected cells is overcome by enhanced degradation of antigen. *J. Exp. Med.* 168 1211–1224. 2459295 10.1084/jem.168.4.1211 PMC2189091

[B59] van de Weijer M. L. Luteijn R. D. Wiertz E. J. (2015). Viral immune evasion: lessons in MHC class I antigen presentation. *Semin. Immunol.* 27 125–137. 10.1016/j.smim.2015.03.010 25887630

[B60] Vattem K. M. Wek R. C. (2004). Reinitiation involving upstream ORFs regulates ATF4 mRNA translation in mammalian cells. *Proc. Natl. Acad. Sci. U.S.A.* 101 11269–11274. 10.1073/pnas.0400541101 15277680 PMC509193

[B61] Verjans G. M. Remeijer L. Mooy C. M. Osterhaus A. D. (2000). Herpes simplex virus-specific T cells infiltrate the cornea of patients with herpetic stromal keratitis: no evidence for autoreactive T cells. *Invest. Ophthalmol. Vis. Sci.* 41 2607–2612. 10937573

[B62] Vyas J. M. Van der Veen A. G. Ploegh H. L. (2008). The known unknowns of antigen processing and presentation. *Nat. Rev. Immunol.* 8 607–618. 10.1038/nri2368 18641646 PMC2735460

[B63] Wang J. Y. Zhang W. Roehrl M. W. Roehrl V. B. Roehrl M. H. (2021). An autoantigen profile of human A549 lung cells reveals viral and host etiologic molecular attributes of autoimmunity in COVID-19. *J. Autoimmun.* 120:102644. 10.1016/j.jaut.2021.102644 33971585 PMC8075847

[B64] Warnock M. G. Goodacre J. A. (1997). Cryptic T-cell epitopes and their role in the pathogenesis of autoimmune diseases. *Br. J. Rheumatol.* 36 1144–1150. 9402857 10.1093/rheumatology/36.11.1144

[B65] Wei J. Kishton R. J. Angel M. Conn C. S. Dalla-Venezia N. Marcel V. (2019). Ribosomal proteins regulate MHC class i peptide generation for immunosurveillance. *Mol. Cell* 73 1162.e–1173.e. 10.1016/j.molcel.2018.12.020 30712990 PMC6697054

[B66] Weinzierl A. O. Maurer D. Altenberend F. Schneiderhan-Marra N. Klingel K. Schoor O. (2008). A cryptic vascular endothelial growth factor T-cell epitope: identification and characterization by mass spectrometry and T-cell assays. *Cancer Res.* 68 2447–2454. 10.1158/0008-5472.CAN-07-2540 18381453

[B67] Whisnant A. W. Jürges C. S. Hennig T. Wyler E. Prusty B. Rutkowski A. J. (2020). Integrative functional genomics decodes herpes simplex virus 1. *Nat. Commun.* 11:2038. 10.1038/s41467-020-15992-5 32341360 PMC7184758

[B68] Yang N. Gibbs J. S. Hickman H. D. Reynoso G. V. Ghosh A. K. Bennink J. R. (2016). Defining viral defective ribosomal products: standard and alternative translation initiation events generate a common peptide from influenza a virus M2 and M1 mRNAs. *J. Immunol.* 196 3608–3617. 10.4049/jimmunol.1502303 27016602 PMC4868770

[B69] Yewdell J. W. Antón L. C. Bennink J. R. (1996). Defective ribosomal products (DRiPs): a major source of antigenic peptides for MHC class I molecules? *J. Immunol.* 157 1823–1826. 8757297

[B70] Yewdell J. W. Dersh D. Fåhraeus R. (2019). Peptide channeling: the key to MHC class I immunosurveillance? *Trends Cell Biol.* 29 929–939. 10.1016/j.tcb.2019.09.004 31662235

[B71] Young S. K. Wek R. C. (2016). Upstream open reading frames differentially regulate gene-specific translation in the integrated stress response. *J. Biol. Chem.* 291 16927–16935. 10.1074/jbc.R116.733899 27358398 PMC5016099

[B72] Zamani B. Moeini Taba S. M. Shayestehpour M. (2021). Systemic lupus erythematosus manifestation following COVID-19: a case report. *J. Med. Case Rep.* 15:29. 10.1186/s13256-020-02582-8 33494816 PMC7832415

[B73] Zanker D. J. Oveissi S. Tscharke D. C. Duan M. Wan S. Zhang X. (2019). Influenza a virus infection induces viral and cellular defective ribosomal products encoded by alternative reading frames. *J. Immunol.* 202 3370–3380. 10.4049/jimmunol.1900070 31092636 PMC6681668

[B74] Zhao Z. S. Granucci F. Yeh L. Schaffer P. A. Cantor H. (1998). Molecular mimicry by herpes simplex virus-type 1: autoimmune disease after viral infection. *Science* 279 1344–1347. 10.1126/science.279.5355.1344 9478893

